# Annotation-free learning of a spatio-temporal manifold of the cell life cycle

**DOI:** 10.1017/S2633903X23000193

**Published:** 2023-10-06

**Authors:** Kristofer delas Peñas, Mariia Dmitrieva, Dominic Waithe, Jens Rittscher

**Affiliations:** 1 Department of Engineering Science, University of Oxford, Oxford, United Kingdom; 2 Big Data Institute, Li Ka Shing Centre for Health Information and Discovery, University of Oxford, Oxford, United Kingdom; 3 Department of Computer Science, University of the Philippines, Quezon City, Philippines; 4 WIMM Centre for Computational Biology, MRC Weatherall Institute of Molecular Medicine, University of Oxford, Oxford, United Kingdom; 5 Nuffield Department of Medicine, University of Oxford, Oxford, United Kingdom

**Keywords:** Cell life cycle, machine learning, self-supervision

## Abstract

The cell cycle is a complex biological phenomenon, which plays an important role in many cell biological processes and disease states. Machine learning is emerging to be a pivotal technique for the study of the cell cycle, resulting in a number of available tools and models for the analysis of the cell cycle. Most, however, heavily rely on expert annotations, prior knowledge of mechanisms, and imaging with several fluorescent markers to train their models. Many are also limited to processing only the spatial information in the cell images. In this work, we describe a different approach based on representation learning to construct a manifold of the cell life cycle. We trained our model such that the representations are learned without exhaustive annotations nor assumptions. Moreover, our model uses microscopy images derived from a single fluorescence channel and utilizes both the spatial and temporal information in these images. We show that even with fewer channels and self-supervision, information relevant to cell cycle analysis such as staging and estimation of cycle duration can still be extracted, which demonstrates the potential of our approach to aid future cell cycle studies and in discovery cell biology to probe and understand novel dynamic systems.

## Impact Statement

Studying the cell life cycle has led to significant leaps in biology and medicine. Here, we describe an approach to extract useful information for cell life cycle analysis using a manifold constructed without the need for exhaustive expert annotations.

## 1. Introduction

The cell cycle plays a critical role in biology and is the subject of many previous studies^(^^1^ ^–^^4^ ^)^ looking at its checkpoints, pathways, and necessary conditions. In studying the different aspects of the cell cycle, morphological changes under certain conditions are observed and analyzed^(^^5^ ^–^^10^ ^)^. However, morphological variations can be subtle, and annotation of single cells can be tedious without automated analysis pipelines.

In recent years, we have seen advancements in microscopy imaging enabling us to acquire data at a larger scale. With this comes the greater need to interpret and to process the large amounts of data in an automated manner. Machine-learning-based approaches have been demonstrated which leverage this large amount of data used in training to build more robust models for tasks such as cell phenotyping^(^^11^ ^,^^12^ ^)^. We have also seen several successes in machine-learning-based cell models to decrease our reliance on annotations and to simplify biological experiment setups while retaining the same level of information that can be extracted^(^^13^ ^)^. With all these, we are now able to fill more gaps in our knowledge of biological processes and more easily revisit, challenge, and update our understanding of known phenomena such as the cell life cycle in light of new discoveries. Gut *et al*. ^(^^14^ ^)^ and Eulenberg *et al*.^(^^15^ ^)^ created independent models that can generate cell cycle trajectories and identify cell cycle stages. CellCognition^(^^16^ ^)^ is an open-source framework that can classify live-cell images into life cycle stages based on their morphology quantified using extracted statistical texture features. Using histone H2B and proliferating cell nuclear antigen (PCNA) markers, a support vector machine (SVM) was trained in a supervised way to automatically label cell trajectories over time. This classification is further refined using a hidden Markov model (HMM). This approach demonstrates a way to include temporal context to resolve ambiguous classifications that arise due to model confusion and the inherently low signal-to-noise ratio of live imaging. Key limitation of this model, however, is the expert annotation requirement to train the SVM and HMM. Aside from being time-consuming, expert annotation requires the explicit discretization of cellular states which may not be suitable in recent efforts to understand the cell life cycle better, beyond what is currently known. Therefore, it is ideal to disentangle cell and cell morphology models from biases that could arise from annotations and our prior knowledge of the cell life cycle, especially in situations where it is perturbed. Newer techniques in machine learning, under the family of self-supervised techniques, that uses label-free modeling are best suited in this regard. DeepCycle ^(^^17^ ^)^ is one example of such self-supervised methods. The method uses classification into four virtual classes based on intensity as an auxiliary task to train a convolutional neural network to encode cell images into a four-dimensional latent space. Projecting the points on this latent space into a two-dimensional plane reveals cyclic trajectories as cells undergo the life cycle. While this approach presents well-formed trajectories in the manifold, it still requires a form of discretization during training due to the arbitrary number of virtual classes. Moreover, the setup requires four channels: brightfield and Hoescht to train their model, and FUCCI channels to create the pseudo-labels. DeepCycle exploits the wide usage of the FUCCI setup in cell cycle studies. FUCCI uses the phase-dependent nature of replication licensing factors Cdt1 and Geminin. Cdt1 (amino acids 30–120) is fused with the fluorescent protein monomeric Kusabira-Orange 2 (mKO2) to serve as a marker for G1 and Geminin (amino acids 1–110 or 1–60) is fused with the fluorescent protein monomeric Azami-Green 1 (mAG1) to observe the S, G2, and M phases. The combination of these two probes, their replication and degradation over time, reliably visualizes the cell cycle stages. The disadvantage of using the FUCCI setup is that two markers are already allotted to characterize the cell cycle. This means two fewer channels available for use to look at specific cell regions and processes tied to the cell cycle. DeepCycle attempts to free up this need for two dedicated channels by estimating the cell cycle stage from only brightfield and Hoescht. Similarly, in this work, we further this reduction by looking at only one channel to estimate the cell cycle stage.

In this work, we propose a different approach based on self-supervised techniques for manifold and representation learning to analyze the cell life cycle. Representation learning covers a family of techniques to encode latent representations of the data that can facilitate the extraction of useful information on the whole data population or between sub-populations^(^^18^ ^)^. Applying representation learning on the analysis of cell cycle data can be advantageous as representation learning has been shown to work in other domains, both biological^(^^19^ ^,^^20^ ^)^ and non-biological^(^^21^ ^–^^23^ ^)^, and some of its key principles, such as minimal supervision and abstraction, can tie perfectly with data-driven scientific research. Using our approach also provides a visualization of the cell cycle using a learned representation that incorporates temporal information from imaging data. This visualization opens up possibilities to interrogate different time points and stages in the cell cycle with minimal input and assumptions.

To construct the cell cycle manifold, we used a combination of generative techniques (generative adversarial networks [GANs]^(^^24^ ^)^ and variational autoencoders [VAEs]^(^^25^ ^)^) to compress high dimensional image data to a lower dimensional space where spatiotemporal patterns in morphological changes during the cell life cycle can be better analyzed and generalized. We highlight the contribution of this work as follows:

1. The learning of the cellular manifold for morphology was disentangled from expert annotation of cell images. This approach in the construction of the manifold based on VAE-GAN readily renders the cell model to discovery and confirmatory studies in a data-driven manner, free from annotation bias and prior knowledge of underlying molecular mechanisms.
2. Even with using only single-channel image data, information relevant to the analysis of the cell cycle can be extracted.
3. A temporal constraint was applied to better structure the manifold for the cell life cycle data.
4. The time-series cell morphology data is represented in meaningful ways that can be used in a broad range of analyses, without the need for supervision and for complex imaging and staining.

## 2. Methods

### 2.1. Model architecture

The model used to construct the manifold of the cell cycle is based on the VAE-GAN architecture^(^^26^ ^)^. The architecture combines a VAE and a GAN with a shared decoder/generator module.

A VAE is a generative model that encodes every data point as distributions in a latent space. From these distributions, we can sample reconstructions of the original input data points. The components of the VAE, the encoder and decoder, are trained jointly such that the output minimizes a reconstruction error (Kullback–Leibler divergence) between the computed posterior and the true posterior distributions.

GANs are another approach to generative modeling. Like a VAE, a GAN is also a generative model in which a generator-discriminator setup is trained in an adversarial manner. The generator network *G* tries to capture the data distribution and samples this estimation to produce artificial data. On the contrary, the discriminator *D* estimates how likely is the sample coming from the training data and not from the distribution in *G.* *G* is then trained to maximize the probability of *D* making a mistake.

**Figure 1.**
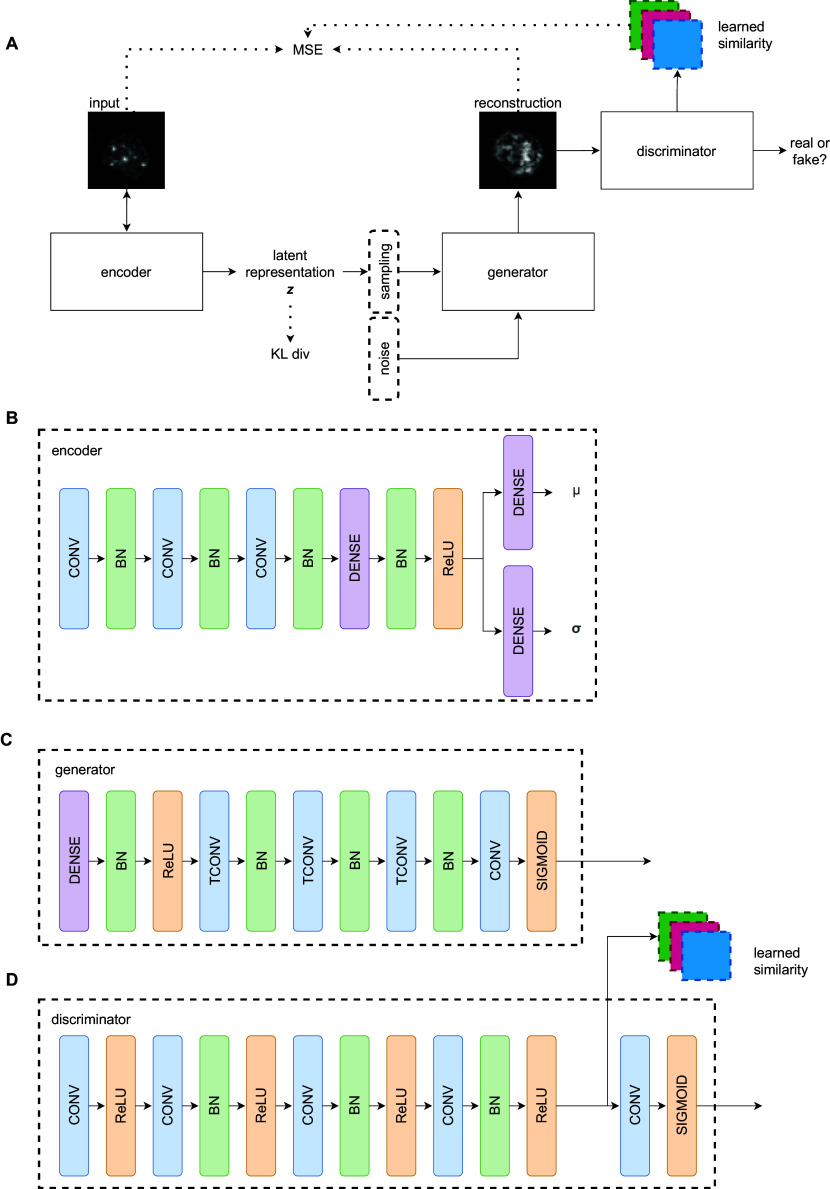
The VAE-GAN architecture used to construct the manifold of cell morphology. The network is composed of three components: encoder, generator, and discriminator. The encoder maps input images to a 16-dimensional Gaussian with diagonal covariance. The generator produces a reconstruction from sampled points in the latent space. The discriminator forces the generator to output images as similar to the input as possible.

VAE-GAN bridges these two techniques and benefits from the advantages of VAE (latent space structure) and GAN (training stability, crisper reconstructions). For this reason, we employed the VAE-GAN architecture to construct the cell cycle manifold. By using a VAE-GAN, we can learn representations that capture the similarities in shape and texture of different cell images from similar time points in the cell cycle. Figure 1 shows the structure of our model. Our model’s encoder contains three convolutional layers with batch normalization, followed by a series of dense feed-forward layers. For all the experiments, the dimension of the latent space is set to 16. The generator/decoder mirrors the encoder with three transposed convolutional layers. For the discriminator, a network with five convolutional layers with batch normalization was constructed. Feature maps after the fourth convolutional layer are extracted and used for the learned similarity metric.

### 2.2. Temporal cycle consistency

Temporal cycle consistency (TCC)^(^^27^ ^)^ is a self-supervised representation learning approach introduced to temporally align similar videos in an embedding space. The approach details a temporal loss based on cycles formed from different corresponding points in a set of trajectories. Let 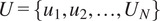 and 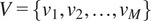 be embedding sequences. In the context of our work, 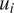 is the latent representation of an image of a cell 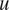 at time point 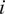 . First, a reference point 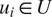 is picked and its nearest neighbor 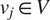 is determined. Next, the opposite is also attempted to be established, that is, 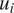 is the nearest neighbor of 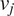 . If both ways hold true, then the pair of points are cycle consistent. The goal is to maximize the number of such point pairs that are cycle consistent. To influence structure on the manifold, however, the process needs to be differentiable. The approach offers a cycle-back classification where each frame in 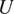 is considered a separate class and reformulates the problem as finding the soft nearest neighbor. In building the manifold for the cell life cycle data, the TCC approach was used to encode the temporal context (ordering of images in sequence) and to improve the alignment of specific stages in the life cycle in the manifold.

### 2.3. Training setup

Training of the model was done in three steps. First, the model was trained to generate good reconstructions of the input images. This is done by setting a lower weight for the KL divergence loss so that the model can focus on minimizing the mean squared error of the input and reconstruction, as well as the learned similarity metric extracted from the discriminator. After this, the second step involves gradually increasing the weight for the KL divergence loss to constrain the latent space representation. This follows the annealing approach described by Larsen et al. ^(^^28^ ^)^ to prevent the collapse of the posterior. Lastly, TCC loss is added to further align the trajectories in the latent space.

All network modules are trained with ADAM optimizers with a learning rate of 0.0001, trained for 1,000 epochs for every step described previously, with checkpoints and an early stopping criterion every 10 epochs. In our experiments, we found that the model converges after 500 epochs in the first two steps of the training process, and after 800 epochs in the last step.

Training and evaluation were done five times to capture the variability of the model performance.

### 2.4. Data

To demonstrate the utility of the VAE-GAN model in the construction of a manifold of the cell cycle, we worked on a dataset from a previous cell cycle study. This dataset is from CellCognition (2010)citeheld2010cellcognition. In this dataset, EGFP-PCNA was used to mark the DNA replication factories and H2B-mCherry for the chromatin of Human Hela Kyoto cells imaged using widefield epifluorescence, 10x dry objective (0.645 μm/px), 1,392 × 1,040 pixel, 482 frames imaged at 5.9 min intervals. In using this dataset, we focused on using only the PCNA channel to build a model that uses only minimal information, further extending the goal of DeepCycle^(^^17^ ^)^ in reducing the marker dependencies in models. This dataset has 408 labeled single-cell trajectories with 255 frames, roughly synchronized at G2-M. We used 60% of these trajectories for training and the remaining for testing. All single-cell images are resized to and centered on 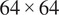 patches, and scaled to the [0,1] range.

## 3. Results

### 3.1. Separation of the cell cycle classes

**Figure 2.**
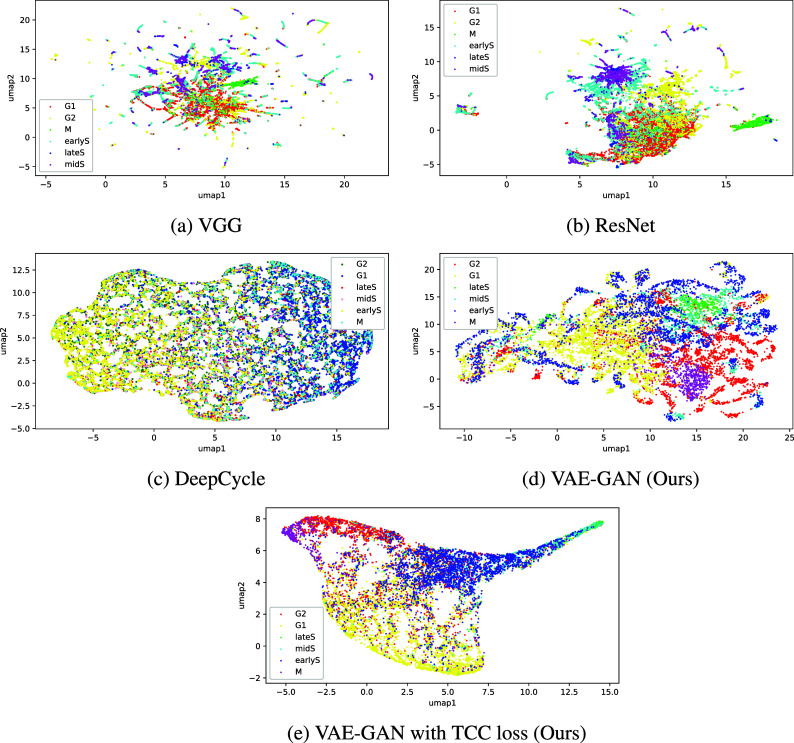
Comparison of constructed manifolds using: (a) VGG, (b) Resnet, (c) DeepCycle, and (d,e) our approach using VAE-GAN. Manifolds from the baseline models (a–c), unlike our VAE-GAN models (d,e), did not produce visually distinct clusters corresponding to cell cycle classes.

We compared the constructed manifold using our VAE-GAN model with manifolds created using baseline off-the-shelf deep learning architectures. The first two baseline manifolds are constructed using the VGG^(^^29^ ^)^ and ResNet-50^(^^30^ ^)^ pretrained models. Feature vectors of size 25,088 and 512 were extracted for each of the images and visualized in 2D using Uniform Manifold Approximation and Projection (UMAP)^(^^31^ ^)^. We plotted the resulting reduced latent points in 2D as shown in Figure 2a,b. In both models, the constructed manifolds did not produce visually distinct clusters of the cell cycle stages. As the VGG and ResNet models are not fine-tuned for cell images and were trained to classify natural image classes, the poor clustering is to be expected and further demonstrates the need for specifically trained model for the cell cycle manifold.

We next looked at an attempt to utilize the technique described in DeepCycle ^(^^17^ ^)^. For this experiment, since the dataset does not have FUCCI channels from which to derive the pseudo-labels for each data point, we tried deriving four intensity classes from the histone H2B channel. We trained the DeepCycle network to output these pseudo-labels in a similar fashion as the original study. However, as can be seen in Figure 2c, the network did not produce good clusters. In the absence of FUCCI markers for the cell cycle, deriving usable alternative pseudo-labels is non-trivial.

**Figure 3.**
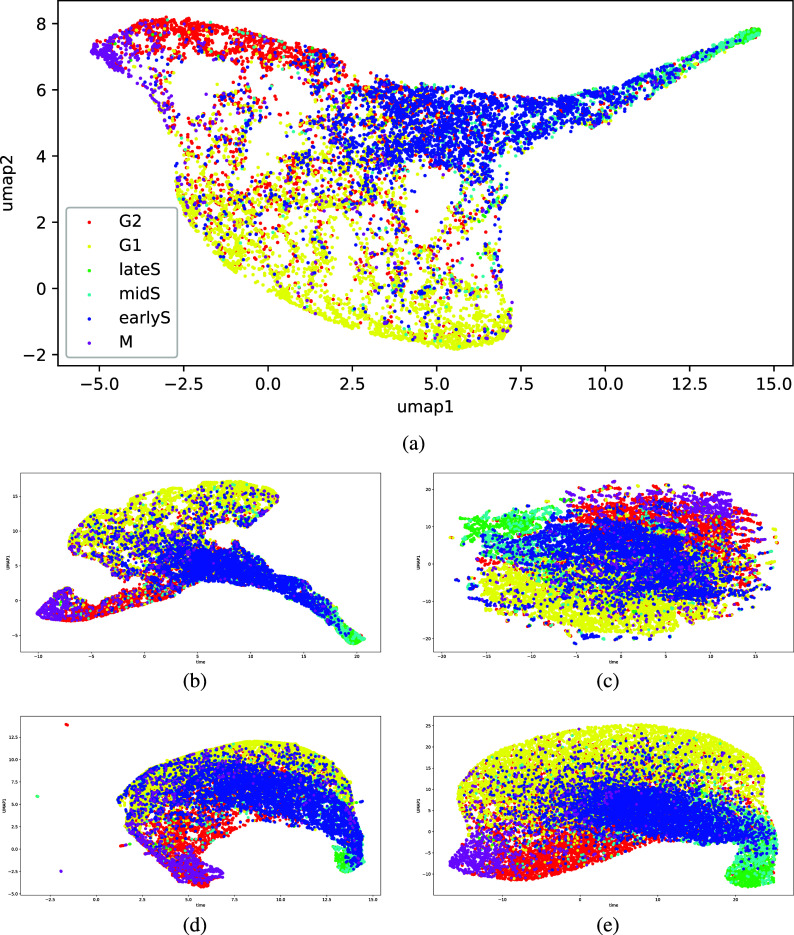
Manifolds across 5 training and evaluation runs. While different after each run, the manifolds constructed show visually distinct clusters for the cell cycle stages.

In comparison, our approach using VAE-GAN resulted in a manifold with more visually distinct clusters. In Figure 2d, even without temporal alignment, we can already observe aggregation of data points corresponding to the M and late S stages. We further improve on this with the addition of the temporal loss in Figure 2e. While there are still cluster overlaps, G1 and G2 now comprise denser and more distinct clusters. Across the five training and evaluation cycles, as shown in Figure 3, our VAE-GAN approach with TCC loss resulted in visually distinct clusters for the cell cycle stages. While each cycle constructed a different latent space, the clusters corresponding to M and late S appear on the extreme ends of the first UMAP axis—an attribute of the latent space that can be useful in downstream tasks.

### 3.2. Cell cycle staging of individual cells

**Figure 4.**
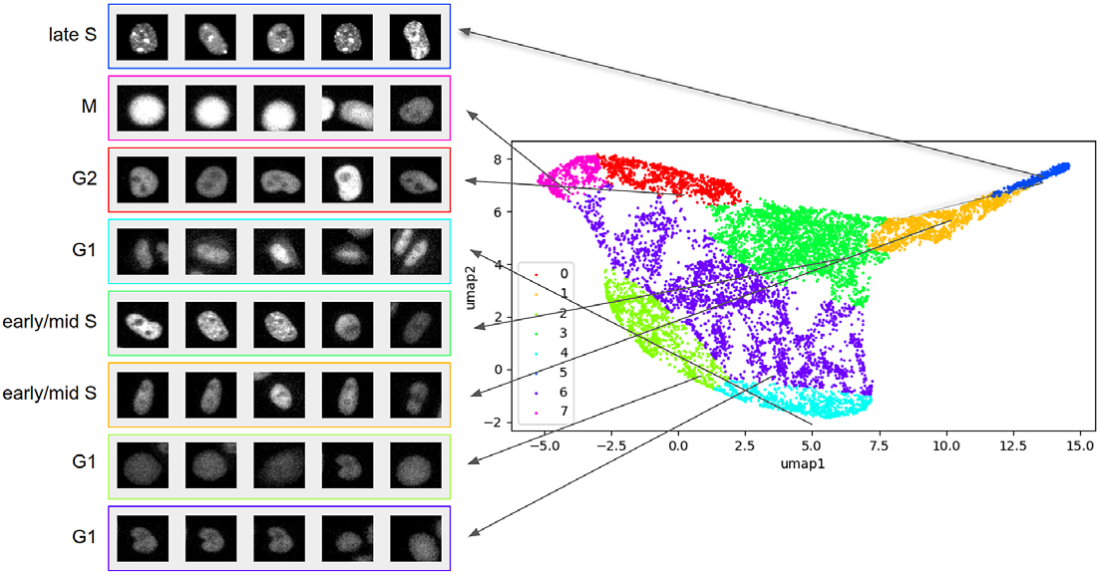
Clusters formed by GMM. Shown on the left are representative raw images from each cluster. Comparing with true labels, the formed clusters roughly correspond to known cell cycle stages: M (magenta), late S (blue), G2 (red), G1 (purple, cyan, light green), and early/mid S (yellow and green). Image size: 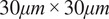 . Contrast and brightness of cell images are enhanced for visualization.

We performed clustering using Gaussian Mixture Models (GMMs) on the latent representations of the data points obtained from the trained VAE-GAN model. In training the GMM, eight components were used. While this number of components is greater than the number of labels in the dataset, the extra components give flexibility in clustering; as we have not optimized for (nor discretized) clusters during training of the VAE-GAN model, the model may identify subpopulations of cell images within a cell cycle stage. Instead of forcing the GMM to output six component Gaussians to correspond to the class labels, we let the GMM form additional clusters that can be manually merged once cluster-class correspondence is established. Figure 4 shows the clusters formed by the trained GMM, projected onto two-dimensions using UMAP. Among the formed clusters, we observed strongly distinct groupings for the late S (blue), G2 (red), and M (magenta) stages. The yellow and green clusters we observed contain both early S and mid-S images. G1 appeared to be spread across three clusters (cyan, light green, and purple).

To validate this clustering, we compared the cluster labels for individual points with the ground truth labels provided in the CellCognition^(^^16^ ^)^ study. The ground truth separates the data into six classes: early S, mid S, late S, M, G1, and G2. We established correspondence of clusters with class labels by looking at the majority of labels within each cluster. Doing so resulted in three clusters matching the G1 class. We merged these clusters to form one G1 cluster and combined two clusters for the early S–mid S (S) cluster, reducing the number of components from 8 to 5. With each component corresponding to a cell cycle class, we evaluated the classification accuracy of the VAE-GAN network using the test images. We extracted the latent representations of these images and used the GMM to label each. With the established cluster-class correspondence, the clustering on the learned manifold resulted in an average of 67.03% accuracy (SD: 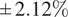 , *n* = 5) across the five training and evaluation cycles. Table 1 shows the confusion matrix for the classification of cell cycle stages.

**Table 1.** Confusion matrix for cell cycle stage classification.

	Without G1-G2 constraint					With G1-G2 constraint				
	M	G1	G2	late S	S	M	G1	G2	late S	S
M	357	212	43	3	46	357	212	43	3	46
G1	13	**3,269**	**46**	7	566	13	**3,290**	**25**	7	566
G2	348	**1,015**	**962**	30	756	348	**94**	**1,883**	30	756
late S	6	12	3	369	59	6	12	3	369	59
S	7	562	103	217	2,974	7	562	103	217	2,974

Values in bold show the changes when the G1-G2 constraint is applied

### 3.3. Identifying key cell cycle stages in cell trajectories

**Figure 5.**
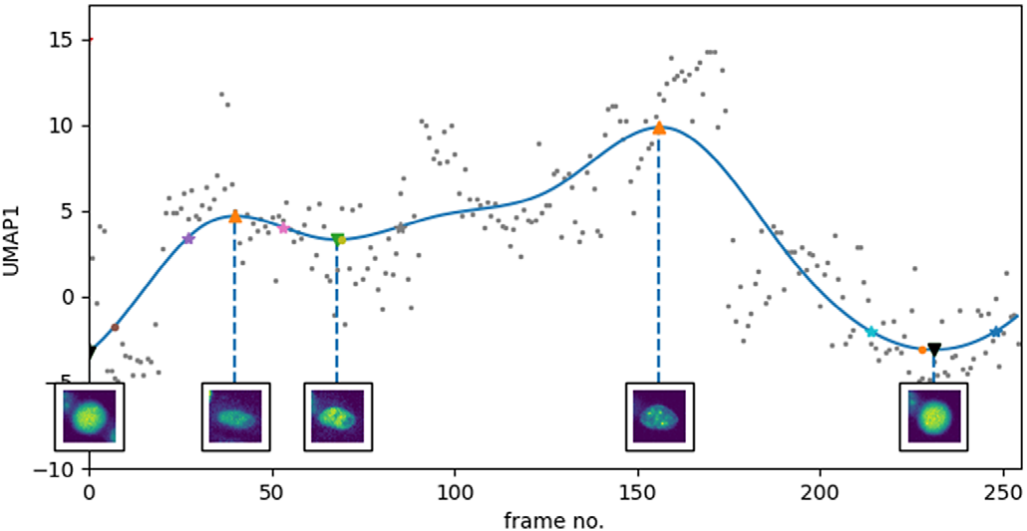
Plotting UMAP1 versus time reveals some key cell cycle stages. Blue line is the smoothed curve using B-splines. Images at timepoints of the crests and troughs of the curve (shown as triangles 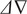 ) are shown and correspond to the mitosis, G1, early S, late S stages in the cycle. Curve inflection points (shown as stars 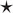 ) where transitions roughly occur are also annotated.

An integral part in the analysis of cell cycle experiments is the staging of individual cell trajectories. In our experiments, we used the constructed manifold to identify key stages in the cycle from the trajectories of the individual cells. While the GMM was able to capture known cell stages as clusters in the manifold, the approach relies on the whole population of cell samples and the number of components to use for clustering. Generally, deciding on the best number of components for GMM is based on some form of prior knowledge. In our case, it was based on the classes available from the previous study and the addition of extra components for flexibility. Another way to look at the manifold is from a single-cell perspective. To do this, we drew trajectories in 2D plots using the first and second UMAP dimensions versus time. While projections in 3D (2 UMAP + time) may be more visually appealing, following single-cell trajectories in 3D would suffer from rough curvatures and oscillations as the manifold was learned without any smoothing constraint for single-cell trajectories. For this reason, we looked at projections of trajectories on the first and second UMAP dimensions (UMAP1 and UMAP2) with respect to time. We observed that plotting the trajectories of cells with respect to the UMAP dimensions is enough to extract some information that can be useful for staging. Utilizing UMAP1, we identified the peaks and troughs of each trajectory—each can be mapped to a key cell cycle stage. Analogous to the Waddington epigenetic landscape^(^^32^ ^)^, these peaks and troughs correspond to known cell states and the ascent or descent would correspond to transitionary states in between. Figure 5 shows a UMAP1 plot for a single cell. In the figure, mitosis stages marking a full cycle appeared as the two lowest troughs in the curve. From the first trough, the curve ascends to the first peak which corresponds to the G1 stage. The curve continues towards another trough that shows early S. It proceeds towards the highest peak (late S) and descends to another deep trough (M). The same trend appears across the other four training and evaluation cycles (inverse trend in one cycle). As shown in Figure 3, the five manifolds share the attribute of having M and late S on either ends of the UMAP1 axis. This demonstrates consistency of the results using our approach.

As G1 and G2 are separated by the S stages, we can enforce a constraint to distinguish between the two growth stages: growth stage that follows S is G2. As we can infer roughly when late S occurs in single cell trajectories using our approach in Section 3.3, we improve on the classification accuracy across our 5 training and evaluation cycles from 67.03 (SD:% 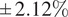 , *n* = 5) to 73.98 (SD:% 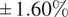 , *n* = 5), or around 75% of the supervised model’s accuracy. We do this by correcting the model’s prediction for G1/G2: any predicted G1 occurring after the inferred time for the late S will be reclassified as G2, and any predicted G2 before the late S will be reclassified as G1. In Table 1 we see a significant change in the confusion matrix after applying this constraint. Initially, 1015 from the G2 class were misclassified by our model as G1. Similarly, 46 G1 data were misclassified as G2. Applying the G1–G2 constraint brings down the number of misclassifications to 94 and 25 for G2 and G1, respectively. The addition of this constraint brings the accuracy closer to that of the fully supervised two-channel CellCognition model.

### 3.4. Estimating cell cycle duration

**Figure 6.**
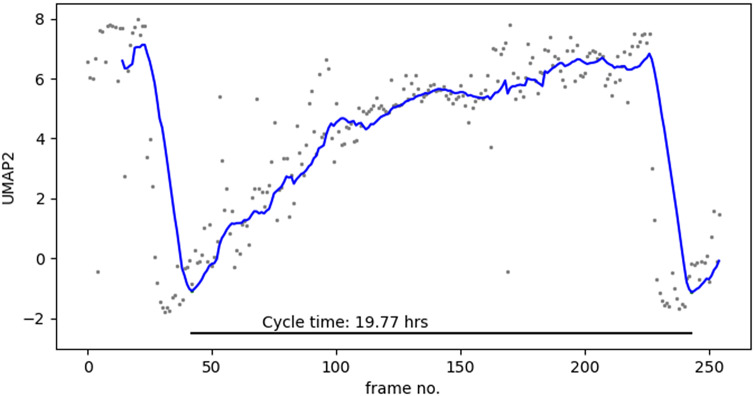
Plotting UMAP2 versus time reveals the oscillating nature of the cell cycle. Blue line is the 10-frame running average. Cycles are marked end to end by sudden drops in the running average curve.

Similarly, we can use the patterns in individual cell trajectories to estimate the duration of a cell cycle. We plotted the UMAP2 value of each data point with respect to time and extracted the troughs in the curve. We expect a repeating pattern. As the cell continues to grow and undergoes S, and G2, it traverses the UMAP2 axis until peaking during mitosis. This is then followed by a drastic drop to another trough, representing the start of another cycle. Using the smoothed curves for each cell trajectory, we can then quantify the cycle time by computing the period of the cycle. Shown in Figure 6 is a UMAP2 plot for a single cell.

For each trajectory, composed of 255 images which totals about 25 hours of imaging, we identified two troughs and computed the time difference between the two as our estimate of the cell cycle duration. Using our approach, we estimate the cell cycle time of a trajectory shown in Figure 6 to be 19.77 hours. Comparing with the cycle time that can be computed in CellCognition SVM model (time between two mitosis classifications), we estimate the cycle time using our approach with root mean squared error (RMSE) of 18.80 minutes or about 2% difference.

## 4. Discussion and Conclusion

Here, we present a self-supervised approach to construct a manifold for the cell life cycle. While several studies developing machine learning models for the analysis of the cell cycle had been proposed in the past, we revisit the topic and propose an alternative approach based on generative modeling and manifold learning.

Our approach consists of a VAE-GAN model trained with an additional temporal loss to encapsulate not only the spatial information in image data but also the temporal context available in live imaging as cells progress through their life cycle. The approach requires only the DNA replication factory marker (PCNA) and does not use an exhaustive form of annotation during training. This disentanglement from complex experiment setups and expert annotation of data can aid discovery and confirmatory studies for the cell cycle as analysis can be free from annotation bias and prior knowledge of underlying molecular mechanisms.

The results from using our approach for common tasks in the analysis of the cell cycle show that even with only the PCNA channel and self-supervised training, relevant information can be extracted from live cell data. We demonstrated two ways by which the data can be viewed and analyzed using our VAE-GAN manifold. First, we can look at the manifold as a means to describe the whole cell population across all timepoints. In this way, we can characterize the variations within and between cell cycle stages. We capture this similarity and dissimilarity through clustering on the latent points encoded by the VAE-GAN model. Our results show that the VAE-GAN manifold forms better-separated clusters than manifolds constructed from the baseline models. From these formed clusters, we then classified and staged individual cells and observed a 73.98% accuracy (SD: 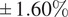 , *n* = 5). Although this accuracy is lower than that of the supervised CellCognition model, the advantage of our approach lies on the non-reliance on annotations and pixel-wise segmentation. Looking at single cells is the second way to utilize the learned manifold. In this way, we can analyze the dynamics of cell morphology during the cell life cycle. Through tracking over time, we can extract time estimates of cell cycle duration and identify some key stages in the cycle’s trajectory. These unveiled information can benefit various live cell imaging experiments that depend on the occurrence of or are focused on specific cell cycle stages. As our proposed approach is self-supervised and incorporates temporal information, it can be a suitable analysis tool for such experiments on the cell cycle. We further envision that the same approach can also be applied to the analysis of other dynamic cell processes such as cell differentiation and cell-cell interactions.

## Data Availability

Data used are from CellCognition study (https://cellcognition-project.org/). Intermediate and analysis files, and code can be made available upon request from the authors.
